# Necrosis

**Published:** 1879-07

**Authors:** R. T. Henderson

**Affiliations:** Shawneetown, Mo.


### NECROSIS.

By R. T. HENDERSON, M.D., Shawneetown, Mo.

[Abstract of a paper read before the South-East Missouri Medical Association.]

Bone, by a peculiar and admirable arrangement of its nutritive supply, is endowed with a grade of vitality equal to that of the most favored tissues of the body; also with all the attributes of vitality, not only in its power of growth, but of development, regeneration, repair and disease.

That bone holds a position among the higher tissues is abundantly vindicated by the variety, frequency and severity of its diseases. We find no morbid condition of the soft tissues of which a counterpart may not be found in bone.

When, through violence or inflammation, the periosteum is detached from the bone, by the interposition of effusion, necrosis is brought about by cutting off the requisite supply of blood. In this way many superficial necroses are produced and also a considerable portion of the thin exfoliations we so often meet with after slight injuries.

Another primary cause of necrosis is found in the non-expansibility of bone-tissue. The interruption of one or more vessels conveying blood to soft parts is very easily amended by the dilatation of inosculating vessels, but in bone the inosculating vessels cannot dilate with the same facility, owing to the unyielding character of the long canals through which they are transmitted. The increased flow of blood in the part produced by the stimulus of inflamation crowds the unyielding capillaries so urgently that transmission of the current becomes slower and slower—the thinner parts of the blood move on, while the corpuscles become more and more jammed and packed in the channel, now relatively too small to receive their increased number, until presently the current is arrested altogether and the circulation ceases. This peculiarity of the circulation would naturally lead us to look for necrosis more frequently in the compact tissue of bone than in the cancellous, which accords with clinical experience.

Location of the bone predisposes it to injury. Miller says, the bones most liable to suffer are those most exposed to atmospheric influence and mechanical violence. "The tibia enjoys an unenviable prominence in this respect;" next he places the femur, then the humerus, cranium, lower jaw, clavicle, ulna, etc.

The most unequivocal of the predisposing causes are constitutional, including those enfeebled conditions of the system which are understood to favor mortification, such as the state induced by long exposure, privation and hardships, and particularly the condition following severe and exhaustive diseases, which diminish the power of the circulation to maintain itself against the sudden assault of inflammation.

The predisposing conditions existing, the disease may be excited by exposure to wet and cold or injuries of any kind. Markoe says, as a direct and immediate cause of death, injury does not often act, but rather by its setting up inflammatory actions, which, by unfavorable influences, shall be so modified as ultimately to produce a fatal effect; and thus we may consider violence rather as the indirect and secondary than as the direct and immediate cause of necrosis.

In support of this I remark that even in compound comminuted fractures we do not expect necrosis, and, on the other hand, under certain unfavorable conditions, a moderate injury will be the starting point of severe inflammatory action, rapidly terminating in necrosis.

Bell relates many authenticated cases of necrosis of the cranial bones, arising from violence so light as not to interrupt the continuity of the soft parts; some cases even arising from forcibly pulling the hair of the head.

Mercury is one of the prime causes of this disease. Prof. Miller says that there is no more frequent cause than imprudent and unnecessary mercurialism. The action here is more frequently that of caries than of necrosis, mainly because the alveolar rather than the compact tissue of the bone is attacked. Markoe says we do, however, occasionally find that the inflammation creeps along the periosteum, separating that membrane from the bone, and producing actual necrosis, generally of a limited portion of the jawbones.

Phosphorus acid, generated in the process of the manufacture of matches, is a very frequent cause of a peculiar form of necrosis of the lower jaw; and it is very remarkable that the operatives having sound gums and teeth are rarely attacked, whilst those who have carious teeth, or spongy gums, are extremely liable to be attacked; and any who are exposed to the phosphorus fumes soon after the extraction of a tooth are almost certain to suffer.

Properly speaking, we cannot say acute and chronic necrosis, yet the periostitis, or osteo-myelitis, which leads to local death, may be acute or chronic—the most extensive and destructive depending on the acute. The greater part of necrosis—that is, the separation of the dead portion of bone and the formation of its substitute—is invariably chronic.

The extent of necrosis is various. Varieties may be classed as superficial, internal, or central, complete—where the whole thickness but not the whole length of the shaft is involved—and total, where the entire bone has perished. This latter is rare. To illustrate this subject I will introduce a case coming under my observation. During the winter of 1860, S. H. S., aged eighteen, a large, robust, healthy young man, free from scrofulous or syphilitic taint, whilst at play with his fellow students, fell over a log, injuring his leg; he limped away, however, and soon thought himself all right again, walked home in the evening—a mile—assisted in doing the chores usually done about a farm house at this time of the year, ate his supper and retired at the usual hour, seemingly well. During the night he was awakened by a severe pain in his thigh, attended with high inflammatory fever. A neighboring physician was called in, who diagnosed acute rheumatism, and conducted the case for several weeks. At the expiration of this time, the patient being greatly reduced, night sweats and other symptoms of exhaustion having set in, another physician was called in consultation, who detected fluctuation, lanced the limb at several points, recommended, instead of antiphlogistics, cod-liver oil, iron, eggs, beef tea, and stimulants. Under this treatment the patient gained rapidly. After a time he began going on crutches, by-and-by threw them away; today he is active and the picture of robust health, yet his leg has never ceased to suppurate for any length of time, and at times has been quite painful, though it is yearly improving. Although I have advised an operation, he has persistently refused.

Now, we will suppose, in this case, that the injury produced an acute periostitis and osteo-myelitis, resulting in necrosis of the femur, of the third variety, that is, involving the whole thickness, but not the entire length of the diaphysis. The periosteum and medulla have suppurated; within the bone, the pus falls to detritus, or actually putrifies ; the pus from the periosteum has been let out through the skin ; the circulation in the diaphysis has ceased, and of course is no longer associated in any of the vital changes which go on about it. The next step in the process is that of separation between the living and dead bone. This action is accomplished by an interstitial proliferation of granulations of the living bone, by which a slight amount of it is consumed, so that at last the osseous substance is entirely replaced by soft granulations at the ends ; the granulations forming here break down somewhat, soften to pus, leaving the ends of the sequestrum entirely detached from living bone. Long previously, however, months and perhaps years before the complete separation of the ends of the sequestrum, its entire circumference is surrounded by a large abscess-cavity, the walls of which consist of tissue infiltrated with plastic matter, and on its inner surface, next the sequestrum, like any other abscess-cavity, it has a granulation layer, which constantly produces new pus. The sequestrum with the detachment of the ends, lies loose in this pus-cavity, imbedded in pus granulations, which, usually pretty accurately, fill up the space between the sequestrum and the abscess-walls.

During the detachment of the sequestrum, other important changes have taken place in the surrounding tissues. These processes consist in an ossification springing up from the parts surrounding the dead bone, reaching from the living bone above to living bone below, which, by its abundance and perfect organization, supplies support. The question arises as to the origin of the new bone, and there are few questions upon which opinions have been more fluctuating and contradictory. According to Billroth, the involucrum is formed in the thickened layer of the wall of the pus-cavity, which, he insists, consists of tissue (connective tendinous, or even of muscle) infiltrated with plastic matter, and not of periostrum, as other surgeons and pathologists contend.

This long envelope continues to increase in thickness, and in the course of years, if sequestrum remains, it may be over half an inch thick, compact and strong. This process applies to a complete as well as a partial necrosis of the diaphysis, and corresponds to that stage in the process of the disease, at which the patient, whose case I have just stated, has arrived and at which he may remain for years to come. But should he get rid of the sequestrum which he is continually carrying in this bony case, the cavity would fill up with granulations, and these would ossify, and the bone be thus completely restored, at least, for all the purposes of practical utility.

Pathologists would do well to enlighten us in regard to the texture of this new structure. It is, especially, strange that no one can tell us positively whether the medullary canal is perfectly restored.

Where the sequestra are small they may be completely absorbed, or they may find their way to the nearest surface to be discharged. But where the sequestrum is large, the mechanical obstruction offered by the involucrum can only be overcome by an operation.

The changes just described also apply to the flat and short spongy bones, except that new formation is much less and entirely wanting. I may here also notice two very striking peculiarities in cranial necrosis : an indisposition to the separation and casting off of the dead bone, and a disposition to spread slowly and gradually, so as to invade large tracts of neighboring healthy bone, thereby rendering this a formidable affection.

The disease, during the earlier stage—the stage of inflammation—is attended with high fever, delirium existing sometimes during the first days. The first exudation probably takes place between the periosteum and bone ; it is here also that suppuration begins, and separating the periosteum from the bone, distends that membrane as far as its unyielding nature will permit, causing intense pain ; by and by, the periosteum gives way, the pus escapes into the soft tissues, and finally finds its way to the surface, when the first stage may be regarded as terminated.

On the evacuation of the pus the fever and pain abates, the swelling subsides, and the patient and—often the physician—congratulate themselves that the case is rapidly returning to a healthy termination. Instead of this, however, it is found that the abscess does not heal ; fistulous openings continue to discharge pus ; the limb remains somewhat swollen and tender, and is liable to occasional recurrence of inflammation. As the case progresses, the swelling and induration of the soft parts gradually disappear, while a deeper and firmer enlargement takes their place, which is manifestly due to the gradual formation of the involucrum. The limb remains but little changed during the time of the detachment of the sequestrum, or as long as the sequestrum remains. The abscess contracts down to mere fistulæ, the soft parts resume their natural condition, and the discharge, though under favorable circumstances not large, continues while the sequestrum remains.

The diagnosis during the first stage is difficult. The diseases with which it is most liable to be confounded are erysipelas, simple phlegmon, or rheumatism, and no one can with positive certainty, in all cases of inflammation, say which are to terminate in necrosis ; but the possibility of the symptoms depending on periostitis, or osteo-myelitis, leading to necrosis being borne in mind, it is not likely that the careful observer will long be deceived.

After the more active stage has passed away, on account of the continued discharge of pus and increased thickness of the extremity, we suspect extensive new formations of bone. The probe, however, will give us definite information. We pass as large a one as possible through the opening from which the pus flows, and, if it be necrosis, we come in contact with the sequestrum, the surface of which is usually smooth and firm, and if detached movable.

Caries is almost the only disease for which necrosis at this stage can be mistaken. The mode of origin and locality aid greatly in the distinction, for necrosis occurs, more frequently, as a result of acute inflammation in the hollow bones, caries usually occurring more slowly in spongy bones. In caries there is but little formation of new bone about the ulcer, often none can be felt ; in necrosis this is extensive ; in caries the pus is thin, bad, sanious ; in necrosis, it is thick, often good, frequently mucuous ; in caries, we pass the probe into rotten bone, and probing is usually quite painful ; in necrosis, the probe generally strikes the sequestrum, and under judicious care is not often painful. Indeed the differential diagnosis between these two diseases is in most cases very simple and easy.

The prognosis in most cases may be rendered favorably ; the conditions most unfavorable being original weakness of constitution or a scrofulous or a syphilitic diathesis.

Necrosis of the cranial bones being the most formidable, is attended with most danger. Aside from constitutional weakness and local danger, we have other sources of danger arising in the progress of the disease, the chief of which is hæmorrhage. We have indicated that the sequestrum when separated has a tendency to work its way towards the surface, during which it is liable to encounter some artery of importance, which may be wounded by its pressure. In long-continued suppurations from bone, albuminuria is not unfrequently developed, and, although usually of a mild form, may be a source of danger.

The first indications in the treatment are to combat the inflammation on which the necrosis depends. This is best accomplished by rest in the recumbent position, painting the limb with strong tincture of iodine, and the administration of saline cathartics. Cold applications are highly recommended.

by some authorities, and are highly to be commended, particularly where the joints become implicated by the inflammation. Some surgeons are in favor of making deep incisions down to the bone, early, or as soon as suppuration begins. Such a procedure I would discourage, because such extensive wounds are bad in feverish patients, and increase the predisposition to pyæmia. Much may be done in the way of securing more perfect quiet and freedom from pain, by means of properly adjusted fenestrated plaster-splints; these are especially commended where the joints are involved. Should our efforts fail in subduing the inflammation, and suppuration occur, as soon as fluctuation can be detected at the thinnest point we should make several openings in such wise that the pus shall escape without being pressed out.

The active inflammatory stage subsiding, the physician has but little to do, except to watch the progress of the disease, until sequestration is accomplished. During this period, if necessary, we prescribe such remedies as will improve the general vigor, and increase the tone of all the active functions of the body, but by no means using chemical or stimulating agents, or mechanical contrivances, with the view of expediting the separation, as recommended by our earlier surgeons; better do nothing, leaving the case to the conservative powers of nature, than to be harmfully meddlesome in our eagerness to do something.

We wait patiently until the sequestrum is *completely* detached, and then remove it. The rule accepted and insisted on is, never to attempt the removal until complete detachment has taken place, a fact not *always* so easily ascertained; yet, in many cases, this can easily be done by the touch of the probe, which immediately reveals the fact if the dead piece is moveable. Where we fail to detect mobility, we will have to trust to probabilities. In such cases, length of time since the bone was ascertained to be dead, the thickness of the bony case, the pouting, exuberant granulations presented at the orifices of the sinuses, are all important aids in determining whether the sequestrum be detached or not. Most sequestra are detached within eight or ten months, the separation taking place earlier in children. Where we are in doubt, it is beeter to postpone interference for a time. Having ascertained, however, that the sequestrum is loose, it is the duty of the surgeon to remove it, by the operation of sequestrotomy.

The first step is, to cut down to the involucrum, selecting the most favorable situation, avoiding important blood-vessels and nerves, and saving the soft parts as much as possible. It is advisable to open on the side on which the fistulous openings present, if practicable. If the opening in the involucrum be sufficiently large, and the sequestrum small, we may seize it with a strong forceps and remove it without trouble. But if the opening be small and the sequestrum large, a portion of the bony case must be removed. Selecting, if practicable, some of the cloacæ as a starting point, with chisel and hammer, gouge, osteotome or panel-saw (some physicians preferring one instrument, others another), we work away carefully, but with never faltering energy, until the opening is sufficient for the removal of the sequestrum with lever and forceps, when the indications shall have been fulfilled. After the operation the cavity is to be kept clean, the patient kept in bed, and great caution exercised for some time. The final filling up of the involucral cavity is a very slow process, and so, therefore, is the final healing of the wound, some cases requiring months and years.

No surgeon is justified in amputating a necrosed limb, unless he has exhausted every resource of our art to save it. In cases where important blood-vessels have become eroded and the hæmorrhage cannot be controlled, amputation may become necessary, or it may be demanded in cases where large joints become implicated in the suppurative process, or in case of extensive death of bone throughout its whole thickness, when the expected reproduction has failed ; or yet, there are, rarely, cases in which the system is unequal to the contest of bearing up under the burden of continued irritation and copious discharge, before the separation of the sequestrum is complete, in which amputation may be demanded to save the life of the patient. But even in these cases a combination of knowledge, judgment and experience should be brought to bear and the great responsibility of sacrificing an important limb should outweigh all other considerations.—*Courier of Medicine.*

